# Training Young Horses: The Science behind the Benefits

**DOI:** 10.3390/ani11020463

**Published:** 2021-02-09

**Authors:** Alyssa A. Logan, Brian D. Nielsen

**Affiliations:** Department of Animal Science, Michigan State University, 474 S. Shaw Ln, East Lansing, MI 48824, USA; bdn@msu.edu

**Keywords:** equine, bone, cartilage, tendon, race, exercise, growth, career, injury, development

## Abstract

**Simple Summary:**

Of common debate among equine professionals and enthusiasts alike, is whether entering race training at two years of age is detrimental or beneficial to the animal’s career and growth. This literature review evaluates epidemiological studies to elucidate that two-year-old horses are not at greater risk of injury compared to older horses. Horses which enter race training at two years of age are, in fact, found to have greater earnings and longer race careers. This review also tackles the impact that exercising an animal at two years of age or younger has on bone, articular cartilage, and tendons. Numerous studies on growing animals have found confinement to be detrimental to normal musculoskeletal growth. However, exercise of dynamic nature in moderate distances, such as that attained with pasture access or prescribed sprints, is beneficial to musculoskeletal development and may prevent injuries when entering race training. Based on scientific evidence, the research cited in this review supports the training and racing of two-year-old horses and advises caution in the use of medications such as corticosteroids.

**Abstract:**

Conflicting research and anecdotal evidence have created disagreement among equestrians as to whether two-year-old horses should be trained and raced. The objective of this literature review is to evaluate epidemiological studies, as well as physiological data on equine bone, articular cartilage, and tendons to better determine the impact of training and racing two-year-old horses. The evaluation of numerous studies on the topic provides evidence that a horse which is trained or raced as a two-year-old has a lower risk of injury and better adapted tissues for the rigors of racing. Unfortunately, the current prolific use of pain-mitigating substances in the racing industry does place horses, including young cohorts, at greater risk of injury, and should be used with caution.

## 1. Introduction

In 2018, the Thoroughbred racehorse Justify won the Grade I Kentucky Derby, and then subsequently went on to capture the North American Triple Crown by also winning the Preakness Stakes (Grade 1 Stakes race—GI) and Belmont Stakes (GI). Having never having raced as a two-year-old, social media was filled with opinions that the onset of training should be delayed until horses are more mature. Justify’s win was attempted to be used as evidence of the benefit of not racing until a horse is three years old or older. However, Justify was the first horse since Apollo, who won the Kentucky Derby in 1882, to have won the race without racing as a two-year-old. In the 136 years between Apollo and Justify winning the Kentucky Derby, over 60 horses who were unraced as two-year-old’s have attempted this feat without success [1]. That statistic, by itself, should lead one to conclude that waiting until a horse is a three-year-old to begin its racing career will not help the horse win the Kentucky Derby (GI). The question that remains, is whether waiting until the horse is more skeletally mature is beneficial to the soundness and longevity of a racehorse or other equine athletes.

Rather than rely on anecdotal evidence, opinions, or traditional beliefs, the best way to answer this is to evaluate the scientific literature for evidence based upon epidemiological studies and an understanding of how the skeleton and other related tissues respond to training and mechanical stress.

## 2. Epidemiological Studies

In 2003, Stover published an epidemiological review of Thoroughbred racehorse studies [2]. In this review, it was determined that musculoskeletal injuries are the greatest cause of racehorse turnover. Though she acknowledged that age can be confounded with other factors, she concluded that two-year-old racehorses are not at greater injury risk compared to horses older than two. Specifically, horses which were older than 4 years of age were at greater risk of injury than younger animals. Exercise was also found to be a contributing factor of injury development in Thoroughbred racehorses. Horses which exercised at high speeds over short distances had lower injury incidence compared to horses which performed single, long-distance high-speed exercises. On the other hand, lack of exercise also impacted the incidence of race-related injuries. At tracks in California, Kentucky, and Florida, it was determined that horses which had experienced a lay-up spanning 21 days to 2 months were more likely to suffer a race-related injury than horses which had raced during the same period.

Other epidemiological studies have determined that Thoroughbred and Standardbred horses which entered race training at 2 years of age had more race starts and greater earnings than those who entered training later in life. Regardless of whether the horses entered in race training at 2 years of age actually raced at that age, the positive stimulus of exercise from training provided advantages to those who started training at 2 years of age [3]. Thoroughbred horses which started racing at 2 years of age had more lifetime starts, wins, money earned, and years raced compared to counterparts who did not start racing at 2 years of age [4]. It is difficult to determine if these two-year-old horses had longer race careers due to a longer duration of actively racing, or if they were able to hold up to a longer race career due to early bone adaptation to the demands of racing.

An analysis of the Australian Thoroughbred racehorse population determined that a higher age at first race start increased the risk of retirement from racing [5]. However, the risk of retirement from racing was lower, and the average distance raced was higher with a greater number of starts as a two-year-old. This study sought to determine the relationship between age at first start and the risk of retirement. An inverse association between age at first start and length of racing career was found, with no supporting evidence that racing at 2 years of age increases the risk of retirement from racing.

A recent meta-analysis of risk factors for catastrophic musculoskeletal injury in racehorses was published by Hitchens and colleagues [6]. This meta-analysis determined horse age and exercise to be two of the many horse-level risk factors for catastrophic musculoskeletal injury. Horses racing or starting race training at an older age had greater odds of enduring a catastrophic musculoskeletal injury. Horses with their first start at 2 years of age were at reduced risk of fatal lateral condylar fractures compared to those whose first start was at 3 or 4 years of age. Similarly, the Equine Injury Database from 2009–2018 published by the Jockey Club reported that, per 1000 starts, 1.37, 1.79, and 1.86 fatal injuries occurred to Thoroughbred horses aged 2, 3, and over 4 years of age, respectively [7]. Horses older than two years of age may not be able to adapt to the dynamic strains placed on bone nearly as quickly as two-year-old’s. Most bone injuries are from repetitive use, three or four-year-old horses may have more time to accumulate repetitive damage, with lesser ability for bone repair compared to two-year-old horses [2].

An evaluation of race-day distal limb fracture in Thoroughbreds in Great Britain supports, as many other studies have, the evidence that fracture risk increases with horse age [8]. This study found an association between age and distal limb fracture risk. It was also determined that the risk of a distal limb fracture is higher in a horse’s first year of racing than in subsequent years. This may be due to animal maintenance problems, such as lack of pasture access and use of pain mitigating substances, as well as lack of sprint exercise during early training of horses. Young horses which have not been exposed to sprints leading up to and during race training are ill-prepared for the increased loads of sprinting during their first season of racing, and sensibly are at greater risk of distal limb fracture. Dorsal metacarpal disease affects over 70% of Thoroughbred racehorses in early training. Horses which are at 2 years of age appear to be more susceptible to dorsal metacarpal disease (bucked shins) than older horses. This susceptibility to dorsal metacarpal disease is most likely due to management, and not age, given that bucked shins can also occur at the initiation of training in horses who start training beyond 2 years of age [2,9]. Dorsal metacarpal disease is characterized by stress fractures in the dorsal cortex of the third metacarpal, partially caused by the lag time between bone formation during remodeling, as rebuilding of bone occurs at a much slower rate than the resorption of bone. Two-year-old racing Quarter Horses and Thoroughbreds are often afflicted with dorsal metacarpal disease, likely because they have not been accustomed to the strains of racing as they have been removed from pasture, kept in stalls, and not afforded voluntary exercise at speed [10,11].

Training and racing of two-year-old horses before closure of epiphysial plates (growth plates) has brought much disagreement between equine professionals and enthusiasts. Some individuals have expressed that young horses should not be expected to perform high-speed work before their radial epiphyseal plates have closed. In 1973, the Australian Veterinary Journal published an article by Mason and Bourke determining the relationship between unsoundness in two-year-old Thoroughbreds and closure of the distal radial epiphysis [12]. This study evaluated two-year-old horses with open, intermediate, or closed epiphyseal plates. At the end of their two-year-old season, 77% of horses that started with open epiphyseal plates remained sound, while 55–56% of horses with intermediate or closed epiphyseal plates remained sound. The authors stated, “Many horses with open epiphyses raced six or more times and remained sound while numerous horses with closed epiphyses became unsound before their first race or before completing six races”. It was also noted that horses with closed epiphyses showed greater incidence of lameness and poor performance. The authors attempted to justify their findings, which were in contrast to common belief, by suggesting that the horses with early closure of epiphyses may have had an unknown nutritional factor that caused a “generalized skeletal dystrophy”. However, it is recommended that children, who just like young horses have cartilaginous growth plates, participate in high-cyclic repetition activities to benefit bone and joint health. Inactivity can be detrimental to epiphyseal growth plate health, as activity is crucial for longitudinal bone growth. Cartilage, like bone, will atrophy without mechanical stimuli from exercise [13]. Given this study, it can be concluded that training and subsequent racing before the radial epiphyseal plates are closed can lead lower incidence of lameness.

Overall, epidemiological studies and the Jockey Club’s Equine Injury Database lend support that racing as a two-year-old is beneficial to the career and health of the horse. While the evidence listed above is sufficient to support racing and training at two years of age, research focusing on tissues affected by exercise should also be considered.

## 3. Bone

Bone is a diverse tissue, which has functions involving locomotion, soft tissue support and protection, storage of minerals, and containment of bone marrow [14]. Wolff’s Law dictates that bone can adapt to its environment, and the strains placed on it [15]. While bone may be perceived as static, it is constantly changing through two similar, yet separate processes: bone modeling and bone remodeling. Bone modeling is the process of bone acquirement and removal in growing individuals, while bone remodeling is the process by which old and damaged bone is renewed [14]. Bone modeling and remodeling involve three unique cells: osteoblasts, osteoclasts, and osteocytes.

Osteoblasts are involved in bone formation through the production of bone matrix constituents [14]. Bone matrix is crucial to the structure and function of bone and is secreted from osteoblasts in the form of non-mineralized osteoid which is then mineralized over weeks to form bone matrix. Osteoclasts are tasked with the resorption of bone. Resorption of bone via osteoclasts occurs much faster than the formation of bone through osteoblasts [16]. Osteocytes function as receptors of strain and communicate with nearby osteocytes or cells on bone surface through a network of cellular processes that run through microscopic canals in the bone matrix, known as canaliculi [16,17,18].

The function of bone modeling is to form or resorb bone to alter and maintain bone shape during growth. Modeling adapts bone to better endure the strains it experiences and is most active during growth and development of the immature animal. While the adult skeleton undergoes some modeling, it is not as frequent as in the immature skeleton. For this reason, while horses are growing, their skeletal strength is highly influenced by the strains their bones undergo through daily use and exercise [19]. Short-term dynamic exercise as an adolescent can lead to beneficial changes in bone morphology, increased fracture force, and reduced fracture risk at maturity [20,21,22,23]. Factors in the strain environment of a bone that elicit remodeling responses include magnitude of strain, rate of change in strain, as well as spread of dynamic strain [24,25,26]. A topic frequently discussed in relation to strain and strain rate is the threshold of bone, which has been shown to be at 1000 με or at 0.1% change in the length of the bone [19]. If bone undergoes a load elevated above its threshold, the bone cells are signaled to increase bone matrix by synthesizing new bone through formation modeling. Consequently, if bone experiences a reduction in loading well below the threshold, resorption modeling commences, and bone removal occurs [19]. For this reason, young horses placed in stalls during early training are subjected to bone loss and a higher incidence of injuries may be seen during this time [10,27]. Walking has been shown to lead to deconditioning of bone in previously conditioned horses, as it does not provide a dynamic strain at a threshold to maintain bone content [28]. However, exercise which elicits bone formation, such as sprinting, during early training could negate the loss of bone due to confinement [20,21,23].

Bone remodeling normally exists in a coordinated relationship between bone resorption and formation. The function of remodeling is to renew primary, damaged, or old bone over time. Bone remodeling occurs throughout an animal’s life, including during maturity. Bone resorption and bone formation occur sequentially during bone remodeling, at the same location on the bone’s surface [19]. Bone remodeling involves three stages: resorption, reversal, and formation. Both osteocyte apoptosis and microdamage to bone will signal osteoclasts to a specific location to begin resorption. During resorption, the osteoclasts will absorb old or damaged bone. During reversal, the process of bone resorption is halted in preparation for osteoblasts to form new bone. Finally, formation occurs and osteoblasts lay down new bone matrix until the earlier absorbed bone is replaced [16]. Resorption and formation stages of bone remodeling are not always in equal balance, especially when injury or disease are present in the animal [19]. Osteoclasts remove bone tissue in a time frame of a few days to 2 weeks. Unfortunately, the formation of bone by osteoblasts can take months. In areas of bone under repair, there is a higher possibility of injury, as bone has been removed yet has not been completely replaced [2]. Knowledge of bone modeling and remodeling justifies that exercise during growth is beneficial, if done properly.

Much of the weight of a horse is borne by the third metacarpal (MC III) [29]. Within 9 days of birth, foals can travel up to 10 km/d [3]. Skeletal development of young horses does not lessen after birth, in fact, it rapidly increases. At 6 weeks of age, birth weight is doubled in Thoroughbred foals. At 12 months of age, the horse is 90% of its mature height and 66% of its mature weight, and by 4 years of age growth has completed [29]. The bones of young horses are most responsive to stimuli up to 2 years of age [3]. Exposing the influenceable skeletal structure of young horses to dynamic loading during development could optimize the skeleton through maturity, thus leading to a lower chance of musculoskeletal injury [3,18].

Warden and colleagues [22] determined that cyclical compression induced onto a rat’s forelimb for 7 weeks (wk) during adolescence can lead to maintained bone quality and strength lasting into maturity compared to non-exercised rats. Greater strength during maturity compared to non-exercised animals suggests that exercise while growing can provide lifelong benefits. Hitchens and colleagues [30] determined that Thoroughbred horses, which sustained a catastrophic musculoskeletal injury while racing or training, had eased off racing in the one to two months prior to the injury. The authors hypothesized that the lack of exercise could have been a result of the horse being unable to complete high-speed exercise due to an injury, or that, conversely, the lack of high-speed exercise led to decreased bone density, meaning the horse was racing with weaker, more porous bone.

Young horses in early race training have a greater incidence of bucked shins compared to their older counterparts already racing. The occurrence of bucked shins in two-year-old Thoroughbreds at the beginning of race training may add to the misconception that young horses are not prepared for the workload of training at two years of age [2,9,11]. Typically, speed is not added during early race training, and horses undergo comparatively long bouts of walking, trotting, and slow cantering. However, the traditional “long, slow” work does not increase bone strength. A study by Spooner and colleagues [31] revealed that even five months of endurance training, in which two-year-old Arabians were trained to perform a 60 km endurance test every three weeks, failed to increase bone mineral content of the third metacarpal. Endurance training has the ability to lead to an anti-inflammatory response to training [32], but does not have the dynamic loading capability to illicit a response in the third metacarpal. During the first few months of race training, the young horse’s skeleton has become accustomed to long, slow bouts of exercise and is not prepared for high-speed work and the stalling that normally occurs once training commences, often resulting in bone loss. Not surprisingly, the onset for bucked shins is most frequently at the addition of speed to the training regimen. Numerous studies suggest increasing the frequency of short-distance high-speed work earlier during training and reducing the frequency of longer, slower exercises to avoid the occurrence of bucked shins [9,11].

Relatively few cycles of dynamic loading are needed to illicit beneficial changes to bone. Logan and colleagues [23] recently determined that 71 m sprints performed at least 1 d/wk lead to a 23% increase in the force required to fracture the fused third and fourth metacarpal of young Holstein calves compared to their confined counterparts which endured no sprints.

A compilation of studies which evaluated bone response to exercise in young animals is shown in Table 1.

## 4. Articular Cartilage

Articular cartilage is a thin layer of specialized connective tissue with viscous elastic characteristics which lines the bone ends of synovial joints. It can withstand high cyclic loads with little to no sign of damage or degeneration. The main functions of articular cartilage are to provide a smooth, lubricated surface for articulation and aid the transmission of loads with a low frictional coefficient [39,40].

During joint loading, contact forces will cause an immediate increase in the pressure of interstitial fluid. This local pressure change causes fluid to flow out of the extracellular matrix (ECM); once the load is removed, interstitial fluid flows back into the ECM. As strain on cartilage is increased, it will increase in stiffness. As age increases, hydration of the matrix will decrease and stiffness related to compression will increase. The ECM is developed, maintained, and repaired by chondrocytes, the resident cells of the cartilage [40]. Collagen in articular cartilage provides tensile strength, while charged proteoglycans provide the compressive stiffness [41]. The rate of collagen turnover is slow in mature animals and it is believed that, at 5 to 6 months of age, equine articular cartilage will have reached its mature thickness [29].

Cartilage contains no blood vessels, lymphatics, or nerves, subjecting cartilage to limited healing and repair. Repair of cartilage components is on a lengthy time frame: turnover of proteoglycans, a major organic constituent of the extra cellular matrix, can take up to 25 years and the half-life of collagen is at least several decades. To further add to the limited repair of cartilage, chondrocytes have little ability to replicate and rely on optimal chemical and mechanical environments for survival [39]. Given these circumstances, articular cartilage does not have many intrinsic healing capabilities, meaning lesions caused by traumatic or degenerative events can progress into osteoarthritis [42].

Regular movement of joints and dynamic loads are required to maintain normal articular cartilage structure and function, with inactivity of joints presenting a detriment to cartilage [39]. Strenuous long-term exercise can lead to site-dependent changes in cartilage composition and depletion of proteoglycans. The ability of cartilage to handle exercise may be influenced by the intensity of exercise and the location within a joint [43]. Similar to bone, cartilage is modified by high strain from vigorous exercise. The in vivo observation of cartilage, as well as tendon tissue, is more challenging than bone. For this reason, the biochemical responses of cartilage to exercise are not nearly as well-known as bone [29]. However, there are still studies that help to elucidate the response of cartilage to exercise.

**Figure 1:**
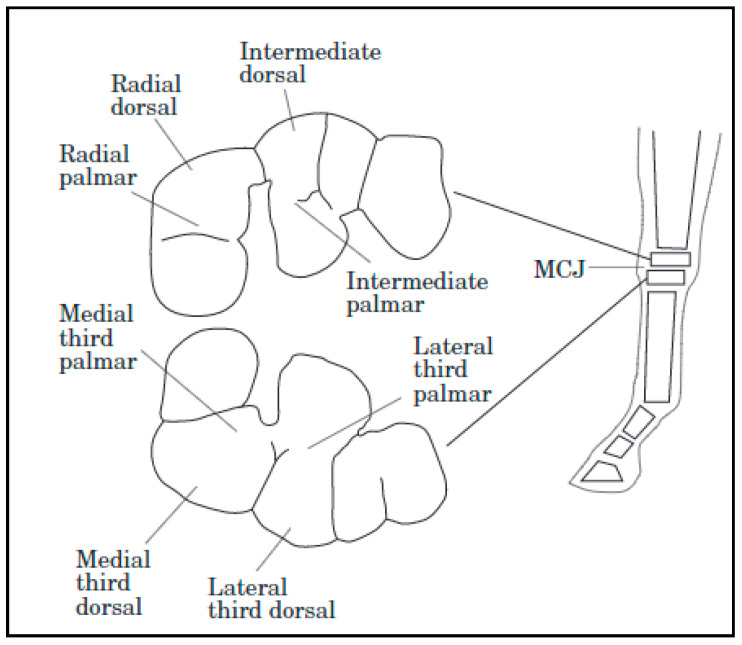
Common sites of cartilage sampling in the carpal joint [43]. Due to the uneven topography of the joint, some sites are predisposed to lesions and alterations to macromolecular characteristics such as collagen and DNA content. Middle Carpal Joint (MCJ).

Thoroughbred fillies of 18–21 months of age undergoing high-intensity training had differences in cartilage biochemical composition when compared to counterparts who had low-intensity training. Collagen content was greater in the dorsal sites than the palmar sites of the high-intensity exercise group. Dorsal sites are exposed to more intermittent loading, while palmar sites bear more constant forces. The high-intensity exercise group also had an increase in total sulfated glycosaminoglycans but demonstrated a loss of homogeneity in distribution through the cartilage depth. The asymmetric distribution of glycosaminoglycans could suggest a loss of proteoglycans or reduced chondrocyte synthesis at high-load sites. Increased glycosaminoglycan synthesis may be a response to exercise; at high-intensity exercise, there could be potential for overloading and loss of proteoglycans in superficial cartilage layers. This study determined that exercise-related differences in cartilage are site specific (Figure 1) [43]. This is because loads are distributed unevenly, and distribution differs based on the gait of the animal [44].

A study published by Van de Lest and colleagues [45] aimed to determine the influence of exercise and confinement on developing equine joints. Horses of 5 months of age, maintained in box stalls with no exercise added to their management, had stunted joint development. Fortunately, the cartilage of young horses is adaptable, and when confined horses were provided pasture access, joint development mirrored that of normal horses. While confinement is detrimental to joint development, over-strain of cartilage can lead to reduced joint development. When young horses were sprinted in excess, between 12–32 sprints of 40 m 6 d/wk for 5 months, deleterious effects on joint health were found, suggesting exhaustion of chondrocytes. The authors have noted that these sprints may have been so excessive that they were considered unnatural exercise to the animals. Pasture access was found in this study to be optimal for joint development compared to confinement and sprint exercise. The forced sprints in this study were far beyond that which has been found to be beneficial to bone. It has been proven that as few as four cycles of loading are needed to maintain bone mass and 36 cycles (a cycle being similar to a stride) are needed to acquire bone mass [24]. A similar study with Thoroughbred foals determined that 1020 m sprints performed 5 d/wk from 10 days of age to 18 months did not lead to a greater Cartilage Degeneration Index of the proximal phalanx of the right hindlimb compared to animals that just exercised freely on pasture. The Cartilage Degeneration Index is a reliable indicator of joint damage, suggesting that sprint exercise did not induce additional wear on joints. The sprint exercise did lead to early maturation in type 2 collagen and glycosaminoglycans of the cartilage ECM. The effects of these biochemical changes are not known to be beneficial or detrimental, nor are the long-term effects of early matured collagen type 2 and glycosaminoglycan in articular cartilage known [46]. The differences between exercise regiments are most likely the cause of the differing results in these two forced exercise studies. It appears that the first study may have required young animals to perform too many sprints, but the second study had animals performing an appropriate number of sprints.

Strenuous exercise of two-year-old fillies who remained stalled except during training lead to disturbances in the collagen network compared to pasture-housed non-exercised counterparts [47]. Two-year-old horses which were strenuously exercised on a treadmill for 19 weeks were found to have thicker calcified cartilage compared to horses who exercised for the same time-period but only at a walk [48]. The author suspected that the greater thickness of calcified cartilage in strenuously exercised animals is an adaptive response to high intermittent loads, as this response was previously noted in canines as well. Conversely, immobilized animals had reduced cartilage thickness. It has been previously determined by Brama and colleagues [49] that confinement during the first 5 months postpartum results in decreased collagen content compared to pasture-kept and forced exercise counterparts. Exercise may need to occur well before two years of age and confinement avoided for cartilage to benefit from exercise.

At this point, it is known that cartilage does respond to exercise as well as confinement and immobilization. Cartilage is also susceptible to injury from too much strenuous exercise [29,39,45]. Bearing in mind that exercise is beneficial for cartilage, while immobilization is detrimental, further studies are still needed to determine the fragile line between exercise frequency which benefits cartilage and that which inhibits cartilage development and function.

## 5. Tendons/Ligaments

**Figure 2:**
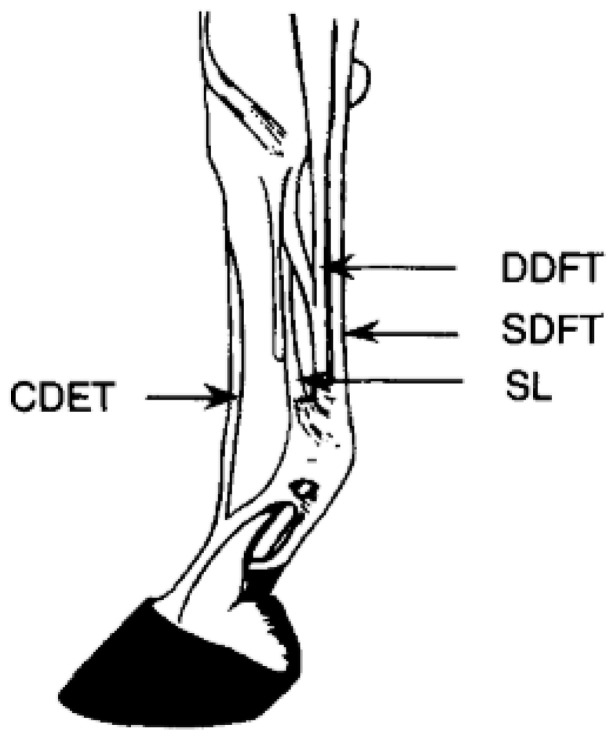
Anatomy of forelimb tendons frequently studied [51]: common digital extensor tendon (CDET), deep digital flexor tendon (DDFT), superficial digital flexor tendon (SDFT), and suspensory ligament (SL).

The functions of tendons include positioning the limb correctly during locomotion and decreasing the energetic cost of motion by acting as springs to store and release energy while stretching and recoiling in the stance and swing phases of each stride [49]. Through acting as biological springs, tendons reduce the musculoskeletal work required at a gallop to nearly half [40]. Tendons are crucial to the transmission of tension from the muscles to the bone [50]. The superficial digital flexor tendon (SDFT) and suspensory ligament (SL) endure greater strains than other tendons and ligaments as they are the main energy storing structures of the horse’s forelimb (Figure 2) [49,51]. As a result of the increased strain placed on the SDFT, it is one of the greatest causes of lameness of Thoroughbred horses in racing and training. Nearly 30% of Thoroughbred racehorses are affected by SDFT injuries [40].

Tendon tissue contains mainly type 1 collagen and has a densely fibrous ECM with high water content [49]. Tenocytes are responsible for the synthesis, maintenance, and degradation of the tendon ECM [52]. Collagen fibrils will be aligned longitudinally in bundles with wavy portions along the fibrils known as crimps. Crimps are buckled during relaxation and will act as shock absorbers which unbuckle during loading. Tendons which have undergone injury will have disturbed crimp morphology and will not respond as well to loading due to the disturbances [53]. Crimp morphology is also a function of age; older animals will have a reduction in crimp angle and crimp length compared to juvenile horses. While tendon fibrils are elastic, tearing occurs if they are loaded beyond the limit they can withhold, and repair of collagen does not occur quickly. Lesions in the SDFT do not typically heal to normal characteristics [54]. Given the poor outlook for repair, prevention of injury would have a greater positive impact than post-injury therapy [52].

The SDFT has limited ability to adapt in mature horses compared to juvenile horses [55,56]. Foals have adaptability in their SDFT and could respond positively to an exercise regimen designed to yield better quality tendons. In horses that were 19 months of age, high intensity exercise on treadmills increased the cross-sectional area of the common digital extensor tendon but not of the SDFT, suggesting that the SDFT was no longer adaptable to exercise in horses of this age [55]. Given the limited adaptability of the SDFT of mature horses, the risk of SDFT injury has been found to increase with age [52].

Loss of homeostasis in the SDFT ultra-structure for up to 72 h after racing has been found to be a normal response to participation in a race for Thoroughbred horses. Another session of maximal exercise is suggested not to occur until the tendon ultrastructure has reached homeostasis again (72 h), as the tendon may be predisposed to injury at this time [56]. Similar to bone, tendon tissue has mechanisms of continual repair and recovery and responds to changes in the strain environment [56,57]. Even with intrinsic repair of tendons, injury requires a lengthy period of recovery, typically up to 18 months [58].

Numerous studies have found that training increases the tensile strength and stiffness of tendons, as well as the cross-sectional areas [57]. In long-distance runners, vastus lateralis tendons are 20% stiffer than those of controls. Increased cross-sectional area of a tendon yields tissue with greater stiffness and reduced strain [54]. Training does have a hypertrophic effect on some tendons, but the SDFT and SL of horses as young as 2 years of age do not appear to increase in cross sectional area in response to training. A response in cross sectional area to exercise could be possible in horses that are even younger than 2 years of age [54].

The SDFT of a foal doubles in cross sectional area from 50 days of age to one year, accompanying the swift increase in body weight. During this time of growth, the tenocyte population is active, but in maturity, tenocytes are significantly less active and collagen turnover is slower [58]. The SDFT reaches maturity before the horse is of 2 years of age, while the deep digital flexor tendon and common digital extensor tendon undergo hypertrophy between 2 and 3 years of age. Exposing horses to training during growth could produce stronger tendons at maturity, especially in the SDFT [51,59]. Foals which were confined to box stalls and sprinted 40 m between 12 and 32 times a day 6 d/wk experienced reduced tenocyte functionality and could have experienced over-stimulation of tendons via exercise. However, in foals isolated to box stalls with no exercise, collagen crosslinks were reduced compared to in exercised animals, suggesting that the tendon maturation process in the stalled foals was negatively affected [60]. Both a lack of exercise and excess of exercise in young horses can impair tendon make-up and subsequent functionality [52,54,60].

Spontaneous pasture exercise may be as beneficial as short sprints performed 5 d/wk from 21 days of age to 18 months of age. Foals with free access to pasture only and foals with free access to pasture along with forced exercise, both experienced an increase in cross sectional area (CSA) of the SDFT but did not experience any differences between treatments. The increase in CSA can be attributed to growth and was greatest during 5–8 months of age, plateauing at about 12 months of age, meaning that the SDFT potentially matures well before the rest of the animal. Ultrasonography found no incidence of tendonitis or injury-related abnormalities in pasture-kept horses or pasture-kept horses with induced-exercise [59]. Unpublished results from this cohort of horses found that there were still no ultrasonographic abnormalities or tendonitis when entering race training at two years of age. If horses do take advantage of spontaneous exercise during pasture access, which young horses often do, developing tendons may benefit and be at lower risk of injury when race training starts.

## 6. Critical Factors to Consider in Injury Prevention

Despite clear scientific evidence that training horses while they are young and growing is beneficial for producing a strong musculoskeletal system that is better prepared to handle the rigors of competition, many in the horse community are quick to condemn this practice. The belief is that waiting until a horse is skeletally mature is the best approach to avoid injury. A recent poll administered on the Facebook page of Feed XL Nutrition Software [61] determined that 79% of their followers believed that horses should start work when almost mature (>4 years of age) and 21% felt that work should start when horses are young (<2 years of age). This survey did not specify racing horses only, and could have encompassed individuals involved in many different equine sports.

In terms of injury prevention, monitoring of training can provide a window into the training process and its impact on the animal. Infrared thermography may be able to detect asymmetries while training Thoroughbred racehorses [62]. This technology could be utilized to detect issues before they become observable. The prevention of injury for a young horse has much to do with owner or trainer flexibility in the horse’s training calendar. Someone who is willing to wait to start a horse until it is four years old, is more likely to be willing to give the horse a rest if a problem develops in training. Individuals who start horses as yearlings or two-year-old’s often “train by the calendar” meaning they have a target date for which their horse needs to be ready to race, and do not allow time for added rest during the season. More experienced trainers in Australia have been found to rest their horses less during a season than their cohorts [63]. While many professionals do start their horses young, they often do not do so with the knowledge that it is beneficial for bone, tendons, and cartilage. Instead, they likely start horses young in order to have them in top performance by the chosen first race date. Thus, when an injury develops, they may be less willing to provide the horse turn-out time and rest to recover from a developing injury, and instead “hide” the injury, be it intentionally, or by using a well-intended treatment that masks the pain but does not cure the injury.

When a problem develops in training, pain-mitigating medications such as corticosteroids are often provided instead of rest or a reduction in training load. Medical administration and management factors also impact the risk of race-related injury. Medical administration is common, with over 70% of Thoroughbred and Quarter Horses in race training in California estimated to be administered medication [30]. Thoroughbreds and Quarter Horses that died as a result of race training had five times greater odds of having hyaluronic acid injections compared to animals that had not died during race training [30]. In Kentucky, horses which sustained a catastrophic musculoskeletal injury had greater levels of nonsteroidal anti-inflammatory drugs [6]. Horses administered a local corticosteroid injection up to 398 days before a race had a greater hazard for musculoskeletal injury compared to untreated horses. Subsequent corticosteroid injections increased the risk of injury. Corticosteroids have potent anti-inflammatory effects but their repetitive use during racing and training alters the articular cartilage mechanical integrity [48]. Though there can be disagreement as to whether corticoid steroids have beneficial or detrimental effects on cartilage, few can argue that corticosteroid administration can result in a rapid decrease in the degree of lameness, presumably by decreasing pain. However, as detailed by Nielsen [64], joint pain is a signal that is useful to warn an animal that care must be taken to avoid causing further damage. When that signal is removed, performance is improved (for instance, faster speeds are achieved) as the animal is no longer protective of the joint, but unfortunately the damage is still present. Even if corticosteroid administration has benefits to joint pain and subsequent performance, articular cartilage cannot be repaired within a matter of days. Thus, it is highly likely that the use of these drugs is a culprit in many of the catastrophic injuries that have been seen on the track. A 2020 article [65] indicated that implementing a 30-day cutoff before races and a 10-day cutoff before workouts on intra-articular injections of corticosteroids in the state of California resulted in a dramatic decrease in catastrophic injuries. While the age of horses entering training has been blamed for catastrophic injuries, the real culprit may be how we manage horses that are developing injuries.

## 7. Conclusions

In young animals, the optimal amount of exercise which is ideal for musculoskeletal strength and performance of function during maturity is not entirely known. Given that this optimal amount of exercise is not yet determined, prescribed exercise during growth is controversial to many. However, it has been shown in numerous studies that confinement and the subsequent lack of loading, lead to weaker tissues and potential loss of function of bone, articular cartilage, and tendons and that exercise during growth aids in the longevity of animal health and performance. Further, medical attempts to decrease pain to allow a horse to train through an injury, instead of providing adequate time to allow an injury to heal, may greatly increase tissue damage—putting horses, and riders at risk.

## Figures and Tables

**Table 1:** Exercise type, and associated bone response in young horses.

Exercise Type	Bone Response	Citation
Trotting	Increased bone mineral deposition when carrying weight compared to no weight	[33]
Endurance training	No alteration to optical bone density	[31]
Sprinting	Greater bone strength and dorsal cortical widths	[23]
	Greater endosteal circumference of third metacarpal	[34]
	Greater bone mineral content and altered bone shape	[20,21]
	Alterations to collagen turnover markers, suggesting lack of collagen synthesis in response to extensive sprints	[35]
Treadmill	Greater impact strength of the third metacarpal	[36]
	Greater radiographic bone density and volume by fraction	[37]
Pasture access	Alterations to collagen turnover markers suggesting less type 1 collagen degradation	[35]
	Greater third metacarpal circumference increased bone mineral content	[38]

## Data Availability

No new data were created or analyzed in this study. Data sharing is not applicable to this article.
